# Features of Celiac Disease in children and adolescents with Down syndrome: a single-center experience of annual screening

**DOI:** 10.3389/fped.2025.1595256

**Published:** 2025-07-17

**Authors:** Martina Lattuada, Paola Rebora, Chiara Fossati, Alessandra Lazzerotti, Lucia Paolini, Alessandro Cattoni, Roberto Panceri, Maria Grazia Valsecchi, Andrea Biondi, Giovanna Zuin

**Affiliations:** ^1^ School of Medicine and Surgery, University of Milano-Bicocca, Monza, Italy; ^2^ Biostatistics and Clinical Epidemiology, Fondazione IRCCS San Gerardo dei Tintori, Monza, Italy; ^3^ Pediatrics, Fondazione IRCCS San Gerardo dei Tintori, Monza, Italy

**Keywords:** Down syndrome, Celiac Disease, children, screening, anti-transglutaminase antibodies, anti-endomysium antibodies

## Abstract

**Introduction:**

Coeliac disease (CD) manifests more frequently in individuals with Down syndrome (DS) and its prevalence varies across different studies. This study aims to assess the prevalence of CD in children with DS and to describe their clinical, serological, and histological features. A secondary aim was to analyze the time needed for the normalization of anti-transglutaminase IgA (TGA-IgA) and anti-endomysium IgA (EMA-IgA) levels in DS compared to non-syndromic (NS) children.

**Materials and methods:**

This retrospective monocentric cohort study included patients with DS under 18 years of age, diagnosed with CD between 2005 and 2022. Each DS patient was matched for year of birth and sex with two NS celiac children. Follow-up was 6-, 12- and 24-months post-diagnosis.

**Results:**

The prevalence of CD in 770 children with DS was 7.5% (95% CI: 5.8%–9.6%). 57 children with CD and DS were compared with 114 CD NS matched controls (total sample size = 171). DS demonstrated less symptoms than 114 NS CD children (26% vs. 79%, *P* < 0.001). In the CD DS group 81% had anti-TGA levels 10 times higher the upper limit of normal, compared to 72% in the control group. Among patients with CD and DS, 93% had histological damage equal to 3rd grade of Marsh-Oberhuber classification at diagnosis. The velocity of normalization of anti-TGA was higher in patients without DS (*P* = 0.005).

**Discussion:**

This study reinforces the higher prevalence of CD in DS, emphasizing the necessity for routine screening, even in asymptomatic individuals. Despite less symptomatic presentation, patients with DS exhibited elevated antibody levels and severe histological damage. Clinicians should expect a prolonged time for antibody normalization following gluten-free diet in DS, mirroring potential challenges in diet adherence and altered immune responses.

## Introduction

Coeliac Disease (CD) is an autoimmune enteropathy triggered by gluten ingestion in genetically predisposed individuals (1). The association between CD and Down Syndrome (DS) has been already extensively assessed; however, prevalence of CD in patients with DS varies considerably across the studies (range 0%–19%) (2–5). In a recent meta-analysis (6) including 4,000 patients with DS the prevalence of biopsy-confirmed diagnosis of CD was assessed at 5.8%, a considerably higher percentage compared to the general population (1% in Western countries) (7, 8). The prevalence of CD achieved 4.6% in an Italian cohort of patients with DS assessed over 20 years ago by Bonamico (9). To the best of our knowledge, no additional data focused on national cohorts have been published subsequently. Moreover, only few studies have assessed the specificities of CD in children with DS compared to non-syndromic (NS) otherwise healthy coeliac children. Furthermore, the time of normalization of IgA antibodies to transglutaminase (TGA) following gluten-free diet (GFD) has been studied in children with CD (10, 11), but no published data are available for patients with DS.

The primary aim of this study was to assess the prevalence of CD in a pediatric cohort of patients with DS and to describe its clinical, serological, and histological features. In addition, we aimed at reporting the DS-specific trendlines of TGA- IgA and anti-endomysium IgA (EMA-IgA) decrease over time, compared to NS children following GFD.

## Materials and methods

### Design of the study

We conducted a retrospective, monocentric cohort study. Clinical records of children with DS under the age of 18 followed by the Pediatric Genetics Outpatient Clinic of Fondazione IRCCS San Gerardo dei Tintori Hospital (Monza, Italy) were retrieved and reviewed by medical staff. All children with DS diagnosed with CD between January 1st, 2005, and December 31st, 2022, were eligible. Coeliac NS age- and gender-matched controls were identified from the Pediatric Gastroenterology Outpatient Clinic of the same Institution. In detail, we selected two NS coeliac patients with superimposable age and gender for each children with DS enrolled. Scheduled follow-up evaluations were performed 6- and 12-months following CD diagnosis and annually thereafter, until the patients turned 18 years. Detailed medical history collection, complete physical examination and CD serology were assessed upon every follow-up visit. The 6–12- and 24-month timepoints were considered for the present study. Informed consent was obtained by parents or legal tutor for each patient. All clinical and laboratorial data were collected and stored in a single excel worksheet anonymously. The study was approved by the Ethic Committee (n° 3896). Results are reported according to the STROBE checklist (12).

### Patients with Down syndrome

In our Institution, from 2005 onwards, all patients with DS underwent an annual work up comprehensive of a follow-up clinical evaluation and laboratory assessment of CD-specific serology. The following data were collected for all patients with DS diagnosed with CD: age, sex, family history consistent with CD, associated symptoms (e.g., variation in appetite, faltering growth, abdominal pain, anemia, constipation, diarrhea, fatigue, vomiting, recurrent infections), anthropometric data (weight, height and body mass index - BMI), concomitant autoimmune disease (hyper/hypothyroidism, diabetes mellitus, psoriasis, vitiligo) and specific serology for CD, ie total IgA, TGA IgA and EMA IgA. When complete IgA deficiency was detected (defined as a total serum IgA level < 5 mg/dl), anti-TG IgG antibodies were considered.

### Patients without Down syndrome

The same clinical and serological data were collected for NScontrols. Specific growth charts for DS (13) and NS patients (14) were used from the CDC (Centers for Disease Control and Prevention).

### Celiac Disease: diagnostic criteria

The diagnosis of CD was made according to the European Society for Paediatric Gastroenterology Hepatology and Nutrition (ESPGHAN) guidelines (15–17) (see Supplementary Table S1). Over the observation period, our laboratory employed different assays to detect TGA-IgA. In detail, the historical Enzyme-linked Immuno Assay (ELISA) method was more recently replaced by chemiluminescence immunoassay (CLIA). On the other hand, EMA IgA have been always detected via immunofluorescent test. Biopsy samples were taken during upper endoscopy from the bulb (at least 1 biopsy) and from either the second or third portion of duodenum (at least 4 biopsies). The procedure was performed under deep sedation. Histopathological findings were classified according to Marsh-Oberhuber classification (18). Endoscopy was performed for all patients with IgA deficiency.

### Statistical analysis

Qualitative variables were represented as absolute number and percentage. Quantitative variables were reported as median and first-third quartiles (Q1, Q3). Mann–Whitney U nonparametric test was conducted to compare the quantitative variables among patients with and without DS and Fisher test for categorical variables. Follow-up time was computed as time between the CD diagnosis to time of last available visit. The time between the CD diagnosis and the time to the first normalization of anti-TGA and EMA was computed to estimate the percentage of normalization by the Kaplan–Meier estimator. Patients lost to follow-up before normalization were censored at their last visit. A multivariable Cox model stratified for period of diagnosis (before/after 2012 to account for the change in the method of detection of TGA-IgA) was also used to evaluate the variables associated with the velocity of normalization of anti-TGA in patients with DS. The velocity of normalization in patients with and without DS was compared by the Wald test with robust Huber sandwich estimator from the Cox model accounting for matched set, with and without prespecified (by a clinical point of view) confounders. Proportionality of hazards was checked by the Schoenfeld residuals. Type I error was fixed at 0.05 and R-cran software (version 4.3.1) was used for the analyses.

## Results

### Study population and prevalence of Celiac Disease in Down syndrome

From January 1st, 2005, to December 31st, 2022, 770 patients with DS were admitted to the Pediatric Genetics' Clinic, among which 58 received a diagnosis of CD, with an overall prevalence of 7.5% (95% confidence interval, CI: 5.8%–9.6%). In the same period, 848 children were diagnosed with CD by the Pediatric Gastroenterology Clinic. Only one patient in the DS group could not be matched by date of birth and sex with any NS, leaving a final sample of 57 CD DS and 114 CD NS controls. Main clinical features of DS compared with NS children were reported in Table 1.

**Table 1:** Main clinical, serological and histology characteristics of celiac patients with and without Down syndrome at diagnosis.

	Patients with Down syndrome (*n* = 57)	Patients without Down syndrome (*n* = 114)	*P*-value
Sex (males), *n* (%)	27 (47)	54 (47)	1
Age at diagnosis (years), median [Q1–Q3]	6 [4.4, 8.7]	5 [3.1, 8.7]	0.05
Diagnosis after 2012 (from 2013 to 2022), *n* (%)	36 (63)	74 (65)	0.866
Family history of Celiac Disease, *n* (%)	5 (9)	42 (37)	<0.001
Symptoms, *n* (%)	15 (26)	90 (79)	<0.001
Diarrhea, *n* (%)	8 (14)	12 (11)	0.614
Growth impairment, *n* (%)	7 (12)	28 (25)	0.071
Abdominal pain, *n* (%)	4 (7)	26 (23)	0.01
Constipation, *n* (%)	3 (5)	9 (8)	0.753
Tiredness, *n* (%)	3 (5)	18 (16)	0.051
Concomitant autoimmune disease, *n* (%)	16 (28)	7 (6)	<0.001
BMI percentile (kg/m^2^), median [Q1–Q3]	50 [(25, 75)]	25 [(11, 70)]	0.414
Serological and histology characteristics			
IgA deficiency, *n* (%)	1 (2)	2 (2)	1
Total IgA * (mg/dl), median [Q1–Q3]	139 [(94, 190)]	96 [(66, 133)]	<0.001
TGA positivity**, *n* (%)	53 (98)	110 (97)	1
EMA positivity ***, *n* (%)	52 (95)	112 (95)	0.331
TGA-IgA ** (ULN), *n* (%)			0.289
0–7	7 (13)	27 (24)	
7–10	3 (6)	5 (4)	
≥10	43 (81)	82 (72)	
Patients undergone EGDS, *n* (%)	42 (74)	105 (92)	0.002
Marsh****			0.558
1–2, *n* (%)	2 (5)	6 (6)	
3a, *n* (%)	8 (19)	12 (11)	
3b, *n* (%)	17 (41)	41 (39)	
3c, *n* (%)	14 (33)	45 (43)	
HLA typing *****, *n* (%)	24 (46)	53 (47)	1

BMI, body mass index; TGA, IgA antibodies to transglutaminase; EMA, IgA endomysial antibody; EGDS, esophagogastroduodenoscopy; HLA, human leukocyte antigen; ULN, upper limit of normal.

*5.8% missing values, **2% missing values, ***1% missing values, ****14% missing values, *****4% missing values.

### Comparison of clinical, histological, and serological features of Celiac Disease in children with Down syndrome and non-syndromic children

In the DS group the frequency of CD in a first degree relative was 9% compared to 37% of NS. Symptoms at time of diagnosis were lower in the DS groups as compared with NS (26% vs. 79%, *P* < 0.001), whereas the incidence of autoimmune disease was higher (DS 28% vs. NS 6% *P* < 0.001). Thyroid disease was the most common autoimmune comorbidity among children with DS (Supplementary Table S2). In DS group, 81% of the anti-TGA levels were 10 times higher than the upper limit of normal (ULN), compared to 72% in NS. An intestinal biopsy sample was taken in 74% of all DS vs. 92% in the control group. Among patients with DS, 93% had histological damage equal to 3rd grade of Marsh-Oberhuber classification while 2 (5%) presented a 1st–2nd grade of Marsh-Oberhuber with anti-TGA levels below 10 times the ULN. In NS patients 6 (6%) presented a 1st–2nd grade of Marsh-Oberhuber (4 with anti-TGA levels below 10 times the ULN and 2 with anti-TGA levels over 10 times the ULN).

Considering the whole sample, the median follow-up was 747 days (first-third quartile 685–802), with a total of 32 patients lacking the 2 years follow-up visit. Supplementary Figure S1 reports the flow of patients among different BMI percentile classes over the 2 years follow-up, showing a reduction of patients in the class of BMI below the 5th percentile in the first year, with a subsequent small increase, without relevant differences among the two groups.

**Figure 1:**
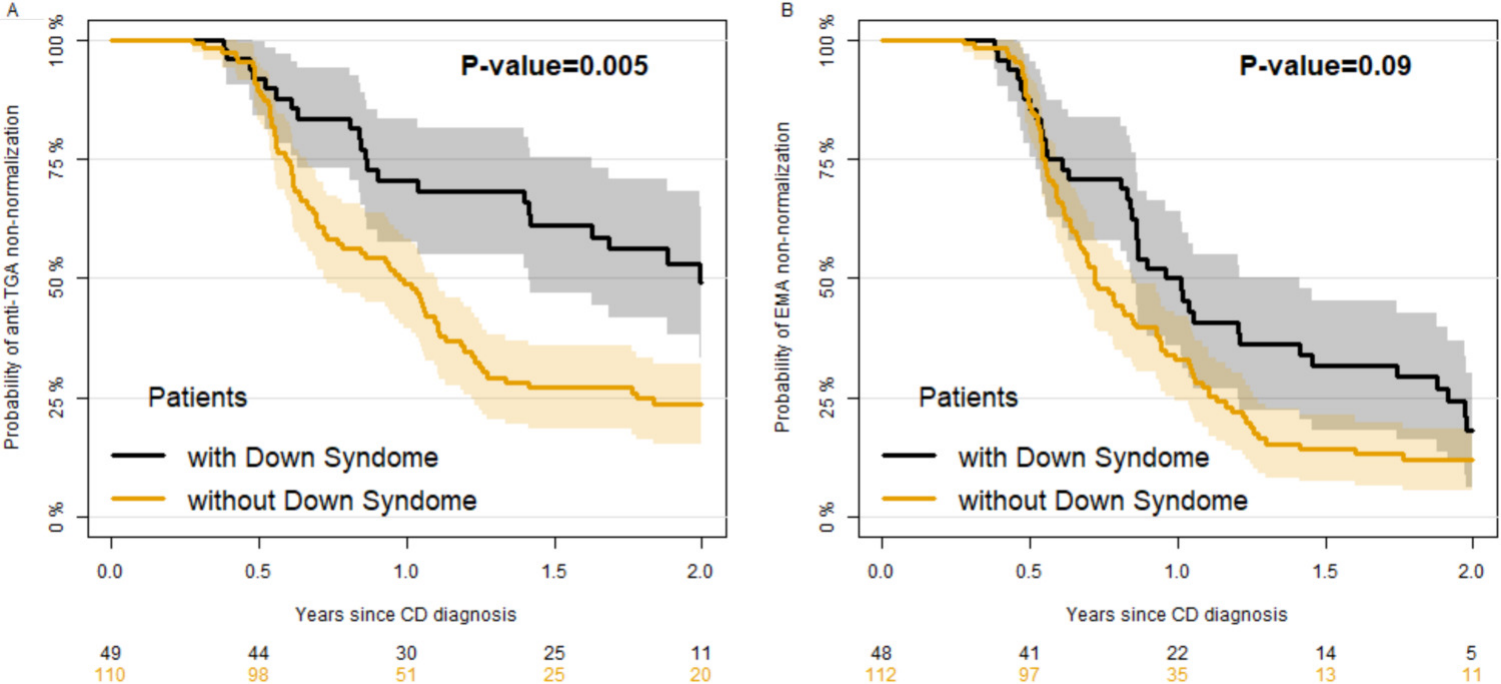
Kaplan–Meier estimate of the percentage of patients positive to anti-TGA (panel **A**) and anti-EMA (panel **B**) since Celiac Disease diagnosis in children with and without Down syndrome.

**Figure 2:**
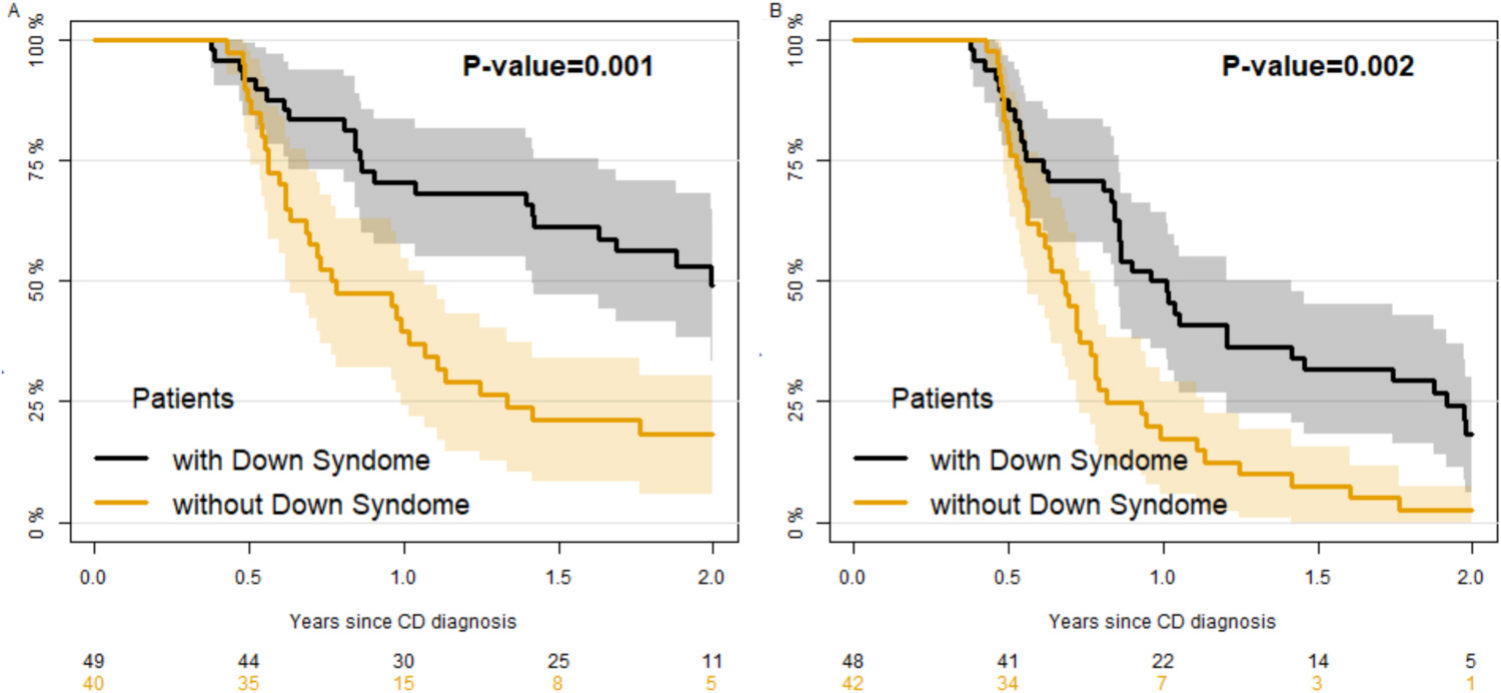
Sensitivity analysis: Kaplan–Meier estimate of the percentage of patients positive to anti-TGA (panel **A**) and anti-EMA (panel **B**) since Celiac Disease diagnosis in children with Down syndrome as compared with children without Down syndrome but with a familiarity for Celiac Disease.

 Figure 1 reports the percentage of children with positive (unnormal) anti-TGA (panel A) and EMA (panel B) by time since diagnosis. The median time to normalization of anti-TGA was significantly higher in DS (727 days, 95% CI 516–805) compared to NS patients (356 days, 95% CI: 263–403) (Figure 1, panel A, robust Wald test *P* = 0.005). The normalization of EMA was quicker, requiring a median time of 370 days (95% CI 308–516) for DS and 263 (95% CI: 239,311) for NS patients (Figure 1, panel B), without a significant difference between the two groups (robust Wald test *P* = 0.09). As children with DS perform yearly screening on CD, while others do not, we performed a secondary analysis selecting a more homogeneous group of controls including only children with a familial CD (and thus who probably had undergone antibody testing for screening). In those controls (Figure 2) the velocity of normalization of both anti-TGA and of EMA was higher, resulting in a significant difference between patients with and without DS (*P* = 0.001 and 0.002 respectively for anti-TGA and EMA). When we evaluated the factors associated with the velocity of anti-TGA normalization (Table 2, model A) in patients with DS, we found that the level of anti-TGA at diagnosis was associated with the velocity of normalization, in particular patients with anti-TGA values higher than 10 times the ULN showed a slower rate of normalization as compared with patients with values lower than 7 times the ULN (HR = 0.05, 95% CI 0.01–0.24). Over the total sample, (including patients with and without DS, Table 2 model B) level of anti-TGA at diagnosis and sex were both associated with velocity, with males being quicker in normalization of anti-TGA. As far as the comparison between patients with and without DS, the normalization of anti-TGA was slower in patients with DS as compared to patients without it (HR = 0.63, 95% CI: 0.40–1.001, *P* = 0.051).

**Table 2:** Results on the Cox regression model on TGA normalization since Celiac Disease diagnosis among children with Celiac Disease with positive TGA at diagnosis.

Variable	Model A on patients with Down syndrome*n* = 49, events = 29		Model B*n* = 159, events = 120	
	HR (95% CI)	*P*-value	HR (95% CI)	*P*-value
Patients with Down syndrome vs. patients without it	–	–	0.63 (0.4–1.001)	0.051
Age at diagnosis (per year)	0.93 (0.8–1.09)	0.374	0.96 (0.91–1.01)	0.142
Sex (males vs. females)	1.52 (0.64–3.58)	0.339	1.53 (1.06–2.2)	0.006
Family history of Celiac Disease (yes vs. no)	1.76 (0.41–7.58)	0.448	1.35 (0.89–2.04)	0.192
TGA-IgA (ULN)				
7–10 vs. ≤7	0.6 (0.08–4.63)	0.626	1.19 (0.5–2.83)	0.641
≥10 vs. ≤7	0.05 (0.01–0.24)	<0.0001	0.39 (0.25–0.62)	<0.0001

CI, confidence interval; HR, hazard ratio; TGA, IgA antibodies to transglutaminase; ULN, upper limit of normal.

Model A includes only patients with Down syndrome (*n* = 49, normalized within the follow-up 29), while model B includes patients with and without Down syndrome (*n* = 159, normalized within the follow-up 120).

## Discussion

In the present analysis, the prevalence of CD in children and adolescents with DS was as high as 7.5%, remarkably greater than the occurrence reported in the historical Italian cohort assessed by Bonamico and colleagues (9). Several reasons may be hypothesized to support this apparent discrepancy, such as a deeper awareness of the clinical spectrum CD, a different serologic approach employed to diagnose CD [antigliadin antibodies (AGA) and EMA by Bonamico vs. TGA and EMA in the present analysis] and the systematic screening strategy introduced in our Centre from 2005 onwards. In addition, despite a patchy geographical distribution worldwide, a growing body of epidemiological studies held in our Country have shed light on the progressive increase of the prevalence of CD in school age children over the last decades (from 0.88% in 1999–2000 to 1.65% in 2017–2020) (19). The data recorded in patients with DS may simply mirror the trendlines of the general pediatric population, though the underlying causes of this phenomenon still need to be clarified.

The question of whether screening for CD is beneficial in the light of a higher incidence among individuals with DS remains a subject of controversy. The ESPGHAN 2020 (17) and the National Institute for Health and Clinical Excellence (NICE) guidelines (20) promote lab screening in populations exposed to a higher risk of developing CD, including DS, but the best timing for scheduled screening remains unclear. Conversely, the American Academy of Pediatrics (AAP) guidelines about health monitoring strategies for children with DS do not recommend systematic screening for CD due to the lack of indisputable evidence about its potential benefits (21).

The prevalence of diarrhea (14%), weight loss (12%), and abdominal pain (7%) observed in this study is superimposable to the findings reported by published literature about patients with DS (9, 22, 23). In some analyses (9, 24), the occurrence of symptoms may be overestimated, as testing for TGA and EMA antibodies were performed exclusively on symptomatic patients. The lower occurrence of clinical findings among patients with DS may be regarded as an expected outcome in a population screened yearly for CD, with the serological diagnosis anticipating the onset of reported symptoms. In addition, gastrointestinal disorders are reported in 50% of children with DS (23), therefore gastrointestinal symptoms may not be reliable indicators for identifying potential cases of CD in this population. In our study, a clinically driven prescription of serological work-up only among symptomatic patients would have led to remarkable underestimation of the prevalence of CD among patients with DS, as only 26% of affected children would have been diagnosed. Accordingly, we find reasonable to suggest a yearly lab screening for CD in DS and to test individuals with new onset of suggestive signs and symptoms.

Even if the BMI percentile median values were unchanged among patients with DS, we witnessed a progressive decrease in the number of patients with low BMI following the prescription of GFD. This data agrees with those published by Nisihara and colleagues (25) on children with DS.

As already extensively reported (9, 22, 26), our study highlights a greater occurrence of autoimmune disorders among celiac patients with DS, compared to NS controls (DS 28% vs. NS 6%). In our study, the most frequently observed autoimmune pathologies were thyroid disorders with a reported prevalence of 19%. The immune dysregulation in DS seems to be correlated with the combination of an increase in the expression of proinflammatory cytokines, an aberrant expression of B lymphocytes and an increase in the production autoantibodies directed against the central nervous system, gastrointestinal tract, pancreas, and thyroid (27).

Regarding family history of CD, our study showed a significantly lower prevalence in the CD diagnosed DS population compared to the control group (9% vs. 37%). To the best of our knowledge, no published studies report the frequency of family history of CD in Down children.

From a diagnostic perspective, 81% of patients with DS showed an anti-TGA titer 10-fold or greater the ULN. Most patients with DS were asymptomatic upon diagnosis and 93% of those undergoing intestinal biopsy showed a severe histological damage. These data are in consistent with the published literature, that reports a poor association between clinically relevant complaints and antibody titers or the degree of histologic damage (28), but a reliable agreement between serological data and histologic damage (28, 29). To the best of our knowledge, our study is the first specifically focused on CD in children with DS.

An additional element of novelty is the longer-lasting time needed to achieve antibodies normalization in DS compared to otherwise healthy controls. Our explanation for this phenomenon is the increased production of autoantibodies targeting the gastrointestinal tract, observed in individuals with DS (27), and the potential difficult adherence to GFD in this population. In a recent work published by Sbravati et al. (11), conducted on the general population, they found a time needed before the normalization of antibodies titer around 9 months from the beginning of the diet, irrespectively of the presence symptoms at diagnosis, with a longer time in those individuals with co-occurrent autoimmune diseases (e.g., diabetes or thyroiditis).

Given its retrospective nature, we are aware of some flaws affecting our analysis. Firstly, we did not have the chance to systematically collect data about the improvement gastrointestinal symptoms following GFD. Moreover, patients with DS were selected through the annual screening, so the probability to detect the antibody positivity before the surge of symptoms was higher than in NS. A multicentric prospective assessment is warranted in order to corroborate our outcomes achieved.

This study confirms a higher prevalence of CD in children and adolescents with DS and highlight the key diagnostic role of CD screening also among asymptomatic patients. Interestingly, even though the majority of DS are asymptomatic and detected through screening, they show higher serologic titers compared to NS controls. Moreover, the average degree of histopathological involvement was superimposable between Down patients and NS controls. This is one of a few works comparing clinical and serological characteristics between Down and non-Down patients. Moreover, it's the first analysis on the antibodies trend since diagnosis in patients with DS. Clinicians should expect a longer time of TGA IgA normalization after the start of a GFD. This should be anticipated to the families to avoid unuseful worries and blood samples to children with DS.

## Data Availability

The raw data supporting the conclusions of this article will be made available by the authors, without undue reservation.
