# Supplementary material for: Features of Celiac Disease in children and adolescents with Down syndrome: a single-center experience of annual screening
Source: Front Pediatr. 2025 Jul 17;13:1595256. doi: 10.3389/fped.2025.1595256 (PMC12310593; doi:10.3389/fped.2025.1595256)
Supplement: Supplementary file 1 [file Table1.docx]

Supplementary Material

**Table 1S:** ESPGHAN 1990, 2012 and 2020 diagnostic criteria for coeliac disease (CD) in children.

| **ESPGHAN 1990** | **ESPGHAN 2012** | **ESPGHAN 2020** |
| --- | --- | --- |
| - Symptoms suggestive for CD - Positivity of IgA antibodies (anti-gliadin, anti-reticulin, anti-endomysium) - Typical mucosal damage at duodenal biopsy - Gluten challenge for children aged 2 years or less | - Symptoms suggestive for CD - TGA-IgA titer > 10 times the ULN - Positive anti EMA-IgA in a second serum sample - Positive coeliac HLA risk alleles DQ2 and/or DQ8 | - TGA-IgA level >10 times the ULN - Positive EMA-IgA in a second serum sample |

Abbreviations: ULN: Upper Limit of Normal; HLA: human leukocyte antigen.

**Table 2S:** Type of concomitant autoimmune diseases.

|  | Patients with Down Syndrome (DS n=57) | Patients without Down Syndrome (nDS n=114) | P-value |
| --- | --- | --- | --- |
| Concomitant autoimmune disease, n (%) | 16 (28.1) | 7 (6.1) | <0.001 |
|  |  |  |  |
| Hypo/Iperthyroidism, n (%) | 11 (19.3) | 3 (2.6) | 0.001 |
| Diabetes mellitus, n (%) | 1 (1.8) | 0 (0.0) | 0.723 |
| Alopecia areata, n (%) | 2 (3.5) | 2 (1.8) | 0.858 |
| Psoriasis, n (%) | 2 (3.5) | 0 (0.0) | 0.209 |
| Vitiligo, n (%) | 1 (1.8) | 1 (0.9) | 1 |

**Figure 1S:** Flow of patients with and without DS among different BMI percentile classes over the 2 years follow-up. Absolute frequencies are reported in the Alluvial plot, while relative frequencies are reported in the table below.


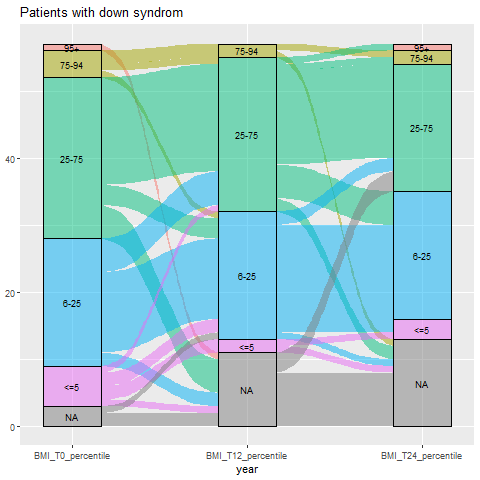

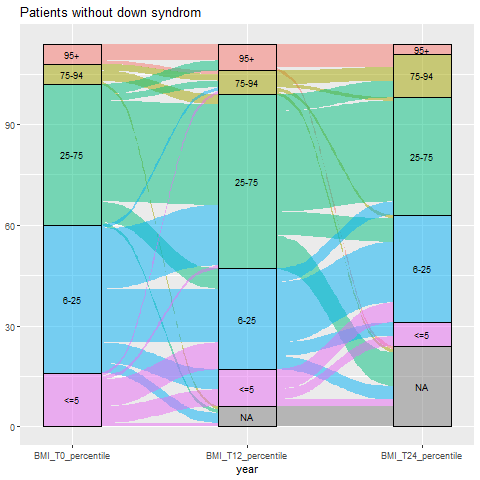


| Time: | Baseline | | 1 year | | 2 years | |
| --- | --- | --- | --- | --- | --- | --- |
| Group: | **DS** | **nDS** | **DS** | **nDS** | **DS** | **nDS** |
| n | **57** | **114** | **57** | **114** | **57** | **114** |
| BMI percentile class (%) | |  |  |  |  |  |
| 95+ | 1 (1.9) | 6 (5.3) | 0 (0.0) | 8 (7.4) | 1 (2.3) | 3 (3.3) |
| 75-94 | 4 (7.4) | 6 (5.3) | 2 (4.3) | 7 (6.5) | 2 (4.5) | 13 (14.4) |
| 25-75 | 24 (44.4) | 42 (36.8) | 23 (50.0) | 52 (48.1) | 19 (43.2) | 35 (38.9) |
| 6-25 | 19 (35.2) | 44 (38.6) | 19 (41.3) | 30 (27.8) | 19 (43.2) | 32 (35.6) |
| ≤5 | 6 (11.1) | 16 (14.0) | 2 (4.3) | 11 (10.2) | 3 (6.8) | 7 (7.8) |
| NA | 3 | 0 | 11 | 6 | 13 | 24 |
| BMI percentile (kg/m2),  median [Q1-Q3] | 50 [25, 75] | 25 [11, 70] | 50 [25, 75] | 50 [25, 75] | 38 [25, 50] | 50 [22, 75] |

Abbreviations: BMI: body mass index; DS: Down syndrome; NA: not available.

**
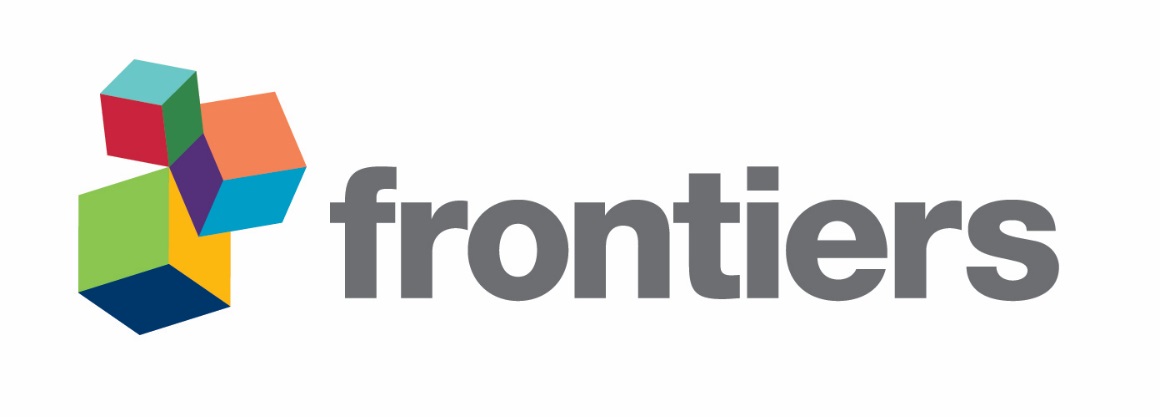
**
